# Effect of male age on semen quality in domestic animals: potential for advanced functional and translational research?

**DOI:** 10.1007/s11259-023-10159-1

**Published:** 2023-07-11

**Authors:** Kenneth Owoicho Abah, Alain Fontbonne, Agnieszka Partyka, Wojciech Nizanski

**Affiliations:** 1 Department of Reproduction and Clinic of Farm Animals, Wroclaw University of Environmental and Life Sciences, 50-375 Wroclaw, Poland; 2 École Nationale Vétérinaire d’Alfort, 94704 Maisons-Alfort, Paris France

**Keywords:** Age, Sperm parameters, Animal, DNA fragmentation, Embryo quality

## Abstract

Age and other factors like season and breed are often associated with sperm quality and fertility in domestic animals. Even though many studies assessed the relationship between the age of the male and sperm parameters, the effects have not been comprehensively evaluated. Changes in semen quality from pubertal (young) to adult and old age were identified in the bull, ram, buck, boar, dog, and stallion, respectively. The review discusses the association between male age and semen volume, the total number of spermatozoa per ejaculate, sperm concentration, motility, morphology, sperm cell function, sperm DNA integrity, oxidative stress, and antioxidant activity in these species of animals. Generally, semen characteristics improve to a certain age, which declines as the animal ages. Only a few studies evaluated the impact of advanced age or employed advanced functional sperm assessment methods to assess age-related changes in sperm quality and male fertility. Such studies in the dog or stallion, for instance, may contribute to advancing knowledge in human-assisted reproductive techniques used in patients of advanced paternal and maternal age.

## Introduction

Numerous studies have reported a correlation between advancing paternal age, semen and embryo quality, sperm DNA integrity, conception rate, adverse pregnancy outcomes, and offspring health in humans (Schwartz et al. 1983; Auger et al. 1995; Levitas et al. 2007; Winkle et al. 2009; Das et al. 2013; Johnson et al. 2015; Halvaei et al. 2020). Conversely, several studies in domestic animals and birds have investigated the influence of male age on sperm characteristics (Hallap et al. 2006; Rijsselaere et al. 2007; Long et al. 2010; Carreira et al. 2017). These studies have revealed that male age can impact various aspects of sperm quality. For instance, in dogs, older males tend to exhibit lower sperm motility and a higher percentage of abnormal sperm (Goericke-Pesch and Failing 2013). Similarly, in bulls, advancing age has been linked to a decrease in semen volume, sperm concentration, and total sperm output (Kelso et al. 1997). However, there is a paucity of information regarding the effects of aging on embryo quality, conception rate, pregnancy, and live birth rate as well as progeny health in domestic animals, in comparison to humans and rodent models. In companion animals such as dogs and horses, genetically valuable male animals are used for breeding even at an advanced age. In these species, particularly dogs, which have high fecundity and litter size, it may be valuable to conduct such studies, especially to assess probable genetic, and epigenetic effects of aging on the health status of the offspring.

Assisted reproduction technologies (ART), such as artificial insemination (AI), have led to improved reproductive efficiency in farm animals (Morgan et al. 2020). The procedure in cattle requires the use of post-pubertal young and medium-aged bulls, from which semen is collected and evaluated for volume, motility, and concentration, as well as for fertility in farms involved in milk programs. Poor-performing bulls are culled, and genetically superior bulls are selected for future AI. One study in which the semen quality of the same bulls at 3, 5, and 7 years of age were evaluated revealed that semen quality at a young age was predictive of semen quality in the older animal (Hallap et al. 2006).

The use of valuable semen for a longer period in farm animals may be important to ensure the genetic progress of the species. However, semen from highly valuable males is not used when they become old and possess poor sperm quality. Understanding the mechanism of reproductive aging can lead to the improvement of the reproductive efficiency of these valuable animals. In this review, we will discuss age-related changes in farm animals such asbull, ram, buck, and boar, as well as in dog and horse, focusing on semen characteristics, sperm DNA integrity, spermatozoa and seminal plasma antioxidant activity, and oxidative stress biomarkers in their semen.

## Aetiologies of age-related decline in semen/sperm quality: overview

**Fig. 1:**
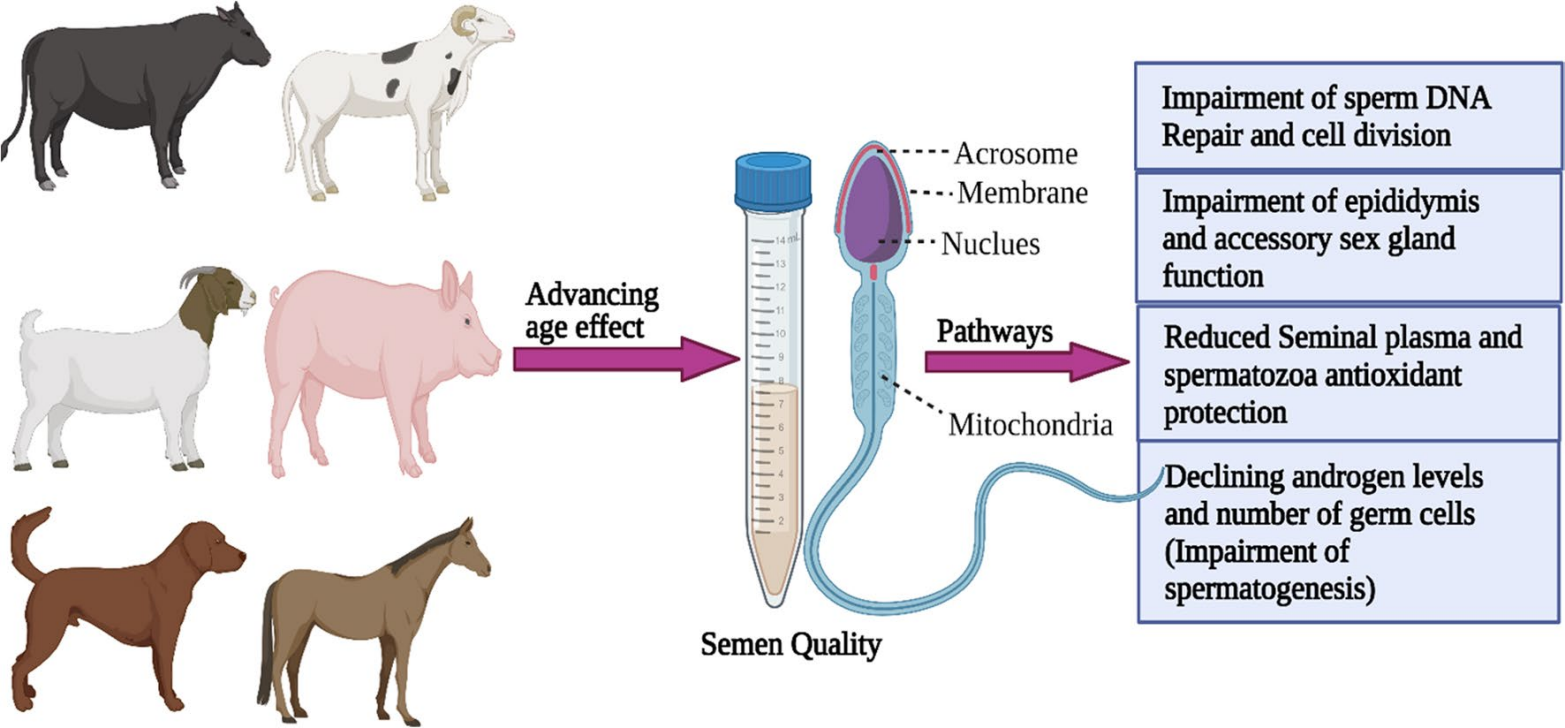
Possible mechanisms of sperm cell aging. Created using https://www.biorender.com

It is well-established that advancing male age is associated with a significant decrease in several semen parameters, such as semen volume, total number of spermatozoa per ejaculate, sperm concentration, motility, morphology, and viability (Winkle et al. 2009; Kipper et al. 2017). Several mechanisms have been proposed to explain the association between these variables (Fig. 1). These factors include decreased antioxidant protection in the semen of aged animals, which may affect sperm viability (Kelso et al. 1997; Carreira et al. 2017); dysfunction of the epididymis and accessory sex glands, which may affect sperm motility (Dowsett and Knott 1996); reduction in the number of germ cells and androgen levels; decreased ability to repair cell and tissue damage as a result of cellular and physiologic changes; systemic and genital diseases associated with aging; and alterations of the male reproductive anatomy, such as decreased blood supply to the testis and constriction of the seminiferous tubules (Halvaei et al. 2020). Based on the available information, it appears that older animals may experience impaired spermatogenesis, which can result in morphological abnormalities in their sperm (Kumi-Diaka et al. 1981; Mandal et al. 2010).

Sperm DNA integrity has been reported to be influenced by increasing male age in different species. The factors associated with decreased DNA integrity may be related to the same etiologies as those affecting sperm characteristics, especially impairment of cell division regulation and DNA repair as animals’ age. As antioxidant capacity decreases with age (Kelso et al. 1997), reactive oxygen species (ROS) may also increase with age, resulting in oxidative stress and DNA damage (Trevizan et al. 2018). In addition, the impact of infection and toxins on sperm cells increases with age, and these are likely to affect the genetic integrity of sperm (Halvaei et al. 2020).

The effects discussed below may result from a combination of these etiologies. However, further studies are necessary to demonstrate a causal relationship.

## Effect of the age on semen quality of the bull

The majority of the studies on the influence of age on sperm parameters in bulls have focused on the prepuberal and adult age ranges, which typically falls within the age range of ≤ 1 to 7 years (Brito et al. 2004; Hallap et al. 2006; Trevizan et al. 2018). Research investigating the effect of advancing age on senior bull (≥ 9 years) fertility is scare, despite the numerous studies concerning the sexual development of young bulls.

It is necessary to state that when investigating the effect of male age on sperm production and quality in bulls, certain confounding factors such as season, breed, and nutrition may affect the outcome. Many studies have attempted to link these confounders with sperm quality at different ages, including Chandler et al. (1985), Mathevon et al. (1998), Fuerst-Waltl et al. (2006), and Trevizan et al. (2018). However, the review did not consider reports on the interaction between age and other factors.

In discussing each of the semen parameters, attention was given to the impact of age on pubertal (≤ 1 year) to senior (≥ 8 years) bulls, pubertal to adult bulls (≤ 7 years), or adult to senior bulls.

### Semen volume, sperm concentration and total number of sperm per ejaculate

In general, semen volume in the bull increases from puberty to adulthood ( Foote et al. 1977; Fuerst-Waltl et al. 2006; Mandal et al. 2010; Bhakat et al. 2011; Murphy et al. 2018; Vince et al. 2018). This increase is typically associated with the development of accessory sex glands, increased scrotal circumference, and increased body weight as bull mature (Ahmad et al. 2011; Boujenane and Boussaq 2013). Specifically, each year of age has been associated with 0.5 mL increase in volume in one study (Murphy et al. 2018). There was an increase in volume up to 4 years (Everett and Bean 1982), 5 years (Mathevon et al. 1998; Bhakat et al. 2011), and 7 years (Taylor et al. 1985) then decline in semen volume was observed.

According to some studies (Everett and Bean 1982; Taylor et al. 1985; Snoj et al. 2013), higher ejaculate volume was observed in senior bulls compared to the young. Similarly, two other studies (Brito et al. 2002; Sontakke et al. 2020) reported higher semen volume in senior bulls than in mature bulls. It should be noted that these studies were conducted on specific populations of bulls and that other factors, such as genetics, diet, and environmental conditions, could affect ejaculate volume.

There are conflicting findings on the influence of age on bull sperm concentration. Different studies have reported varying results (Mathevon et al. 1998; Fuerst-Waltl et al. 2006; Balić et al. 2012; Kipper et al. 2017; Vince et al. 2018).

It has been found in some studies that sperm concentration does not vary significantly after one year of age, indicating that the total number of spermatozoa per ejaculate is primarily due to the progressive increase in testicular production (Everett and Bean 1982; Murphy et al. 2018).

A direct relationship has been identified between paternal age and total number of spermatozoa (TSE) in several studies involving young and adult bulls (Mathevon et al. 1998; Brito et al. 2002; Bhakat et al. 2011; Karoui et al. 2011; Vince et al. 2018). One study found a 33.5% increase in total sperm between young (≤ 24 months) and adult (> 54 months) bulls (Boujenane and Boussaq 2013), and another reported an increase from 5.08 billion spermatozoa at 2 years of age to 6.18 billion spermatozoa at 6.3 years of age (Everett and Bean 1982). Obviously, testicular growth/volume is a major factor in determining total sperm output as it leads to an increase in the amount of seminiferous epithelium. However, this increase is only possible up to a certain age (Boujenane and Boussaq 2013). There was a reduction in the total number of spermatozoa between adult and senior bulls in a few studies (Everett and Bean 1982; Taylor et al. 1985; Snoj et al. 2013). But one study reported a contrasting result where sperm count was higher in senior than adult and young bulls (Brito et al. 2002).

### Sperm motility, morphology and viability

Several studies have shown an increase in sperm motility in adult compared to young bulls (Hallap et al. 2004; Konenda et al. 2020; Mandal et al. 2010). One study reported that Swedish red and white AI bulls at 1 year of age had 56.9% post-thaw sperm motility, while the same bulls at 4 years of age had 69.9% post-thaw sperm motility (Hallap et al.^2004^). Studies involving senior animals (≥ 8 years) reported a decline in sperm motility from adult to senile age ( Kelso et al. 1997; Karoui et al. 2011; Carreira et al. 2017; Kipper et al. 2017; Trevizan et al. 2018). One study recorded subjective motility scores for bulls of different age groups on a scale of 0–5. According to their findings, senior bulls received a score of 2, adult bulls received a score of 4, and young bulls received a score of 5 (Kelso et al. 1997). Sperm motility changes between young and adult bulls represent the stage of sexual maturity and stability, while changes between adult and senior bulls correspond to a period of senile impairments of the testis and epididymis (Carreira et al. 2017).

One Factor responsible for reduced sperm motility in advancing age is decreased antioxidant protection in the semen of aged *Bos Taurus* bulls, which can lead to increased oxidative damage to sperm cells and impair their motility (Kelso et al. 1997; Carreira et al. 2017). Another contributing factor may be impaired function of the accessory sex gland and epididymis, as sperm motility depends on suspension in seminal plasma, and swimming capacity is acquired during maturation in the epididymis (Sartorius and Nieschlag 2010). Additionally, changes in hormonal levels and reduced testicular function can also affect sperm motility in aging bulls.

Interestingly, one study found that although young bulls had significantly higher total and progressive sperm motility than aged (senile) bulls, they showed significantly lower sperm kinematics such as average path velocity (VAP), straight-line velocity (VSL), and curvilinear velocity (VCL) (Carreira et al. 2017). It has been suggested that the spermatozoa of young bulls have lower sperm kinematics due to their immaturity, as they have not yet achieved complete flagellar motion (Carreira et al. 2017). This suggest that as bulls mature, their spermatozoa may develop better flagellar motion, resulting in improved sperm kinematics.

The Percentage of normal spermatozoa has consistently been found to increase from young to medium-aged bulls (Karabinus et al. 1990; Trocóniz et al. 1991; Söderquist et al. 1996; Hallap et al. 2005). This improvement in sperm morphology may be due to factors such as increased scrotal circumference and improved heat control processes as bulls age (Vilakazi and Webb 2004). One study showed that the percentage of abnormal sperm in Holstein Friesian x Sahiwal crossbred bulls decreased as they matured from young to adult. Specifically, the study found that the percentage of abnormal sperm in the head, midpiece, tail, and total decreased by factors of 1.85, 1.27, 1.20, and 1.44, respectively (Mandal et al. 2010). The pattern of sperm morphology changes with age differs from that of sperm motility in that an increased percentage of normal spermatozoa was observed in senile compared to young bulls (Carreira et al. 2017). This was hypothesized to be due to alterations in chromatin condensation in young bulls, which led to increased abnormal sperm head morphology (Kipper et al. 2017; Carreira et al. 2017).

However, some studies have reported no age-relatedeffects on sperm morphology in bulls (Chandler et al. 1985; Ahmad et al. 2011; Menon et al. 2011).

Sperm membrane integrity has been reported to decrease with advancing age, with a significant reduction in senile bulls (≥ 8 years) compared to young (≤ 3 years) (Fuerst-Waltl et al. 2006; Carreira et al. 2017). Some studies have reported a significant improvement in sperm membrane integrity between young and adult AI bulls (Hallap et al. 2004, 2006). However, one conflicting study suggests that plasma membrane integrity (PMI) is not affected by age in Sahiwal bulls. The study found that bulls aged 25–30 months had a mean PMI of 48.3%, while bulls over 100 months had a mean PMI of 49.8% (Ahmad et al. 2011). 

### Sperm chromatin integrity

Sperm DNA integrity can be assessed by evaluating sperm nuclear protamination, sperm chromatin compaction, and sperm DNA oxidation (Carreira et al. 2017). Protamines are proteins found in the nucleus of spermatozoa and are responsible for the packaging and condensation of the paternal genome. Unlike spermatid, the DNA of mature spermatozoa, is compacted and stabilized by protamines. The GC-rich minor grooves of immature spermatozoa are concealed by the arginine-rich sequence of the protamines, as a result, they are inaccessible to chromomycin A3 (CMA-3) binding. The inability of CMA3 to bind the minor groove in the presence of protamines forms the basis for the use of this fluorochrome as an indicator of protamine deficiency (Fortes et al. 2014). Moreover, CMA3 binding is inversely correlated with the level of sperm nuclear compaction (Silva and Gadella 2006).

Carreira et al. (2017) conducted a study involving bulls of Nelore breed and found that the percentage of sperm CMA-3 staining was higher in young bulls (1.57%) compared to adult (1.09%) and aged (0.90%) bulls. Similarly, Fortes et al. (2014) found that the age of bulls was negatively correlated with high CMA-3 binding and positively correlated with low CMA-3 binding. These studies suggest that there is a difference in the level of condensation of the sperm nucleus between young bulls, adult bulls, and aged bulls. Specifically, it suggest that the sperm nucleus of young bulls is less compacted than that of adult and aged bulls. (Table 1).

**Table 1:** Age-related changes on sperm chromatin integrity

Parameters	Species	Effect	References
Protamination	Bull	Defective protamination in young compared to adult and senile	Fortes et al. 2014 Carreira et al. 2017
Chromatin condensation	Bull	High chromatin condensation in adult compared to senile and young	Karabinus et al. 1990 Kipper et al. 2017 Carreira et al. 2017
	Bull	No significant difference	Hallap et al. 2005 Fortes et al. 2012
	Ram	No significant difference	Badi et al. 2018 Ntemka et al. 2019
	Boar	Lower sperm chromatin instability in mature than young	Tsakmakidis et al. 2012
	Dog	Higher DNA fragmentation in old and young than middle age	Hesser et al. 2017
	Dog	No significant difference	Bhanmeechao et al. 2018
Oxidative DNA damage	Bull	Higher in senile compared to young and adult	Trevizan et al. 2018

The chromatin compaction in mature spermatozoa is different from that in other cells in the body. This unique organization of the nucleus results from a complex process that begins in the late stages of spermatogenesis, specifically during spermiogenesis, and is completed during sperm epididymal transit. One study found that the degree of sperm chromatin condensation was not significantly different among young (1.13%), adult (1.06%), and senile (1.04%) bulls when assessed by toluidine blue staining (Kipper et al. 2017). This finding may be due to the limitations of the toluidine blue method in assessing chromatin compaction.. However, the same study found that young bulls had a higher proportion of sperm with larger head area compared to adult and senior bulls. This could be due to defective chromatin compaction, and reproductive immaturity as indicated by CMA-3 staining (Kipper et al. 2017). Additionally, the study did not find evidence to suggest that a higher percentage of sperm with abnormal chromatin compaction levels (4 to 16.15%) is associated with lower rates of successful early embryonic development. This may explain the hypothesis that early embryonic development and fertilization are not affected by bull sperm DNA damage (Fatehi et al. 2006).

DNA fragmentation index (DFI) refers to the percentage of sperm cells with damaged DNA or defective protamination (Fortes et al. 2014). The threshold for DFI percentage varies among species. For example, it is 30% in humans (Evenson et al. 2002), 28% in stallions, and 6% in pigs (Kumaresan et al. 2020). Using the sperm chromatin structure assay (SCSA), a test to evaluate the quality of sperm chromatin, it was found that both young (3.55%) and senile (3.09%) bulls had a significantly higher DFI compared to adult (2.13%) bulls (Carreira et al. 2017).These findings suggest that caution should be exercised when using senile bulls or introducing young bulls early in breeding programmes, as both may have a certain degree of nuclear or chromatin instability, which can affect the quality of sperm and subsequently, fertility (Carreira et al. 2017). A similar study showed that sperm nuclear chromatin of adult bulls was more resistant to acid denaturation after staining with acridine orange (Karabinus et al. 1990). In contrast, an association between chromatin stability and age was not found in another study, although the study had a limited age range. (Fortes et al. 2012; Hallap et al. 2005). However, the study found that spermatozoa with high DNA damage were more likely to have abnormal sperm morphology (Fortes et al. 2012).

It was reported that the percentage of sperm cells with oxidative DNA damage was significantly higher in senile bulls (44.1%) compared to adult (34.9%) and young (25.7%) bulls (Trevizan et al. 2018). They demonstrated that thawed semen of young bulls is more resistant to oxidative stress than adult and senior bulls and showed a positive correlation between lipid peroxidation and DNA damage. Research findings indicate that susceptibility to oxidative DNA damage increases with aging and is associated with reduced antioxidant capacity in semen, insufficient response to reactive oxygen species by testicular germ cells, and failure of the DNA repair apparatus during spermatogenesis (Paul et al. 2011; Selvaratnam et al. 2015; Trevizan et al. 2018).

A study involving Simmental bulls found a significantly lower level of the antioxidant enzyme glutathione peroxidase in the seminal plasma of older bulls (5–10 years) compared to younger bulls (1342.7 U/g protein vs. 1501.5 U/g protein, respectively) (Vince et al. 2018). Similarly, the seminal plasma of Holstein/Friesian bulls, 2–3, 5–6, and > 9 years old was evaluated, and was found that the level of superoxide dismutase was negatively associated with age (Kelso et al. 1997). The spermatozoa of young (1.8 to 2 years), adult (3.5 to 7 years), and senior (8 to 14.3 years) bulls were evaluated and in the level of plasma membrane, it was found a linear increase of lipid peroxidation with increasing age (43. 3%, 49.0%, 53.6%, respectively) (Trevizan et al. 2018).

## Effect of the age on semen quality of the ram

Despite the presence of potential confounders such as breed of sheep, season of collection, and small sample size in many studies, the impact of age on ram semen quality appears to be consistent. The age range considered during most studies can also introduce bias, as only few a studies have used old rams aged 8 or older. Most studies suggest that ram sperm reaches optimum quality at three years of age but begin to decline afterward (Mandiki et al. 1998; David et al. 2007; Hassan et al. 2007; Chella et al. 2017). Interestingly, older rams (≥ 8 years) may have better sperm quality than younger ones (≤ 1 year) (Martí et al. 2011).

### Semen volume, sperm concentration and total number of sperm per ejaculate

Many studies have observed a significant increase in semen volume with age(Wiemer and Ruttle 1987; Hassan et al. 2007; Lymberopoulos et al. 2010; Ntemka et al. 2019). Specifically, one study found a 200% increase in semen volume at 30 months of age compared to 11 months of age in Awassi ram (Salhab et al. 2003). In contrast, other studies reported no significant change in semen volume with age (Toe et al. 1994; Tabbaa et al. 2010; Badi et al. 2018; Benia et al. 2018).

Several studies have reported a positive correlation between male age and sperm concentration in the ram (Salhab et al. 2003; Hassan et al. 2007; Martí et al. 2011; Ntemka et al. 2019), with one study reporting a 36.6% increase in sperm concentration in rams 3 years of age compared to yearling Yankasa rams (Osinowo et al. 1988).

It has been observed that increasing age can influence total sperm output in Lacaune and Manech tete rouse breeds of sheep (David et al. 2007). In one study, total sperm output was found to increase by 68.6% at 3 years of age compared to yearlings (Osinowo et al. 1988). The increase in volume, sperm concentration, and total sperm output per ejaculate was attributed to a larger scrotal size at 3 years of age (Osinowo et al. 1988).

### Sperm motility, morphology, and viability

Several studies have reported an increase in sperm motility with increasing male age in rams (Martí et al. 2011; Chella et al. 2017; Benia et al. 2018; Andreeva and Stefanov 2020). Unlike bulls, the few studies involving old rams (≥ 8)showed that sperm motility in senior rams is significantly higher than that of young rams (≤ 1), and there is no statistically significant difference in sperm motility between mature rams (≥ 3) and old rams (Martí et al. 2011; Ntemka et al. 2019). It was suggested that the hypothalamo-pituitary–gonadal axis of old rams, even up to 13 years of age, is still functioning efficiently,which allows them to maintain good spermatogenesis (Ntemka et al. 2019). In contrast, Osinowo et al. (1988) conducted a study on Yankasa rams and found a significant reduction in sperm motility in rams that were 3 years old. Similarly, Wiemer & Ruttle, (1987) conducted a study on fine wool range rams and also reported a significant reduction in sperm motility at 3 years of age. The discrepancy observed could potentially be attributed to the breed of animals used in these studies. It could be speculated that sperm motility declines with age in certain ram breeds (Wiemer and Ruttle 1987; Osinowo et al. 1988).

 The influence of age on ram sperm morphology has produced inconsistent results, with various studies reporting a reduction, an increase, or no significant change in the number of abnormal sperm cells with age (Wiemer and Ruttle 1987; Osinowo et al. 1988; Mandiki et al. 1998; Hassan et al. 2007; Badi et al. 2018).

It has been consistently found that sperm membrane integrity in rams increases with age (Chella et al. 2017; Ntemka et al. 2019). In one study, Martí et al. (2011) found that rams aged 8 years and above had a significantly higher sperm viability than those aged 1 year and below, with a viability rate of 64.6 ± 1.08 compared to 57.1 ± 0.82, respectively., This finding was proposed to be as a result of larger sperm head area of the young, which may affect its structural and functional competence (Martí et al. 2011).

### Sperm chromatin integrity

Studies regarding the effect of age on ram sperm DNA integrity are few. One study on Chios ram and another on Boujaad rams reported no significant difference in the level of DNA compaction and lipid peroxidation among different age groups (Badi et al. 2018; Ntemka et al. 2019).

## Effect of the age on semen quality of the buck

Several studies have attempted to describe the influence of increasing age on sperm parameters in bucks (Al-Ghalban et al. 2004; Furstoss et al. 2009; Suyadi 2012; Mia et al. 2015; Atara et al. 2018; Hafizuddin et al. 2021; Zaghloul et al. 2021). However, most of the reports have involved only yearlings and young animals, indicating that the effect of aging has not been fully elucidated in this species. This suggests that artificial insemination and selection of animals for improvement of production and reproduction traits are not common in the buck.

Aside from conventional semen parameters, studies in bucks have not extensively investigated functional and biochemical sperm parameters such as acrosome integrity, DNA integrity, mitochondrial potential, apoptosis, lipid peroxidation, oxidative stress, and antioxidant activity.

### Semen volume, sperm concentration and total number of sperm per ejaculate

Reports on the influence of age on semen volume, sperm concentration, and sperm output in bucks are highly inconsistent (Al-Ghalban et al. 2004; Furstoss et al. 2009; Suyadi 2012; Mia et al. 2015; Atara et al. 2018; Hafizuddin et al. 2021; Zaghloul et al. 2021). This inconsistency may be attributed to several factors, such as sample size, breed variation, season, and the subjective nature of the evaluation methods.

Two studies have investigated the semen characteristics of mature bucks up to 6 years of age (Kridli et al. 2005; Gore et al. 2020). In the first study, the authors examined mountain black goats (BG) and BG × Damascus cross bucks. They found no significant differences in semen characteristics between yearlings and mature bucks, except for semen volume (Kridli et al. 2005). In the second study, the authors focused on Saanen and Tuggenburg goats and reported a significant difference in sperm concentration between young (1–2 years) and adult (3–6 years) goats (Gore et al. 2020).

### Sperm motility, morphology and viability

Results on the effect of age on sperm motility, morphology, and membrane integrity in bucks are generally variable.One study involving mature bucks up to 6 years old found an improvement in progressive motility (72.05% vs. 81.30%), mass activity (3.60 vs. 4.12; score 0–5), and consistency (1.97 vs. 2.73; score 0–5) between young and adult bucks, respectively (Gore et al. 2020).

## Effect of the age on semen quality of the boar

Studies on the influence of age on sperm quality in the boar stud are limited to a short time interval of 8 months to 3 years of age, which is shorter compared to other species of domestic animals (Tsakmakidis et al. 2012). The high replacement rate of boars is due to factors such as the need for genetic diversity, poor semen quality, foot and leg disorders, and reduced soundness and libido after 3 years of age (Huang et al. 2010). It is generally observed that, except for exceptional boars, they are typically replaced after their third birthdays (Tsakmakidis et al. 2012).

### Semen volume, sperm concentration and total number of sperm per ejaculate

Generally, the ejaculate volume of young boars (8 to 9 months) is smaller compared to mature ones (up to 3 years), resulting in fewer sperm but with a higher concentration of spermatozoa than older boars (Jankevičiūtė and Žilinskas 2002; Kondracki et al. 2005). One study observed the highest semen volume in various breeds of pigs aged 24 to 29 months old (Kennedy and Wilkins 1984). This finding may be due to physiological development of the testis with increasing age.

One study in different breeds and cross-bred boars reported a rapid increase in sperm output up to 3.5 years of age and then a decline afterward (Smital 2009). This finding suggests the onset of impairment of spermatogenesis and alteration of epididymal and accessory sex gland function after 3.5 years of age.

### Sperm motility, morphology, and viability

The findings on total sperm motility in boars appear to be inconsistent, with different studies reporting either no significant difference with respect to the age of the boar or higher sperm motility in young or middle-aged boars (Kennedy and Wilkins 1984; Šerniene et al. 2002; Jankevičiūtė and Žilinskas 2002; Kondracki et al. 2005; Wolf and Smital 2018).

Many studies have shown that young boars (7–9 months) and adult boars (≥ 3 years) have significantly fewer sperm with normal morphology than middle-aged boars (1–2 years) (Tsakmakidis et al. 2012; Banaszewska et al. 2015). In contrast, a number of studies have also reported a significant increase in sperm morphological abnormalities between young and middle-aged boars (Huang and Johnson 1996; Šerniene et al. 2002; Wolf and Smital 2018). This discrepancy could be due to the influence of confounding factors such as boar genetics, diet, breed, and other environmental factors.

Studies have shown thatthepercentage of live spermatozoa decreases with increasing age. Two studies conducted on Lithuanian white and Pietran pigs, by Kennedy and Wilkins, (1984) and Šerniene et al. (2002), respectively, observed a significant difference in the percentage of live spermatozoa from young and old boars.. In the study by Kennedy and Wilkins, (1984), the percentage of live sperm was found to be 61.9% in boars aged 9–11 months and 59.20% in boars aged 60 months or older.

### Sperm chromatin integrity

To our knowledge, only one study has reported the effect of age on sperm chromatin packaging in boar studs. Using the acridine orange test, it was observed that mature (18–33 months) boars had significantly lower sperm chromatin instability (SCI) (1.62 ± 0.30%) than young (7–8 months; 3.51 ± 0.76%) and old (51–61 months; 3.33 ± 0.70%) (Tsakmakidis et al. 2012). This finding suggest that there are differences in sperm chromatin integrity (SCI) between young and mature boars. Young boars exhibited higher SCI, which may be attributed to abnormal chromatin compaction and larger head area of sperm. This could be indicative of defective protamination, a process where sperm DNA is condensed and stabilized during spermatogenesis. (Tsakmakidis et al. 2012). Older boars exhibited higher sperm chromatin instability, possibly due to increased oxidative DNA damage, which can occur as a result of aging and oxidative stress.

The study Compared the fertility of young, mature, and old boars, and it was found that young boars had a significantly lower farrowing rate (65%) than mature (87.24%) and old (84.74%) (Tsakmakidis et al. 2012). It was hypothesized that the reason why old boars had significantly higher farrowing rate than the young ones, despite having similar values for sperm chromatin instability (SCI), was that higher percentage of live morphologically normal spermatozoa counterbalanced the detrimental effect of SCI (Tsakmakidis et al. 2012). However, the age of the boar stud has been reported to have no significant difference in litter size (Tsakmakidis et al. 2012).

## Effect of the age on semen quality of the dog

Artificial insemination is a widely used technique in small animal reproduction clinics, and the use of fresh and frozen sperm samples are common. However, how aging affects the charateristics of these sperm samples have not been fully explored in dogs. Typically, veterinarians and dog breeders consider dogs between the ages of 1 and 3 as young, 4 to 6 years as middle aged, 7 to 8 years as old, and 9 or greater as senior dogs (Hesser et al. 2017; Fuente-Lara et al. 2019). As dogs age, the quality of their semen can decline, which can impact their fertility. Therefore, it is important to fully explore the influence of aging on the fertility of frozen semen in dogs, in order to provide valuable advice to owners of stud dogs regarding the right age to collect semen for cryopreservation.

### Semen volume, sperm concentration and total number of sperm per ejaculate

Semen volume, sperm concentration, and total sperm output appear not to change significantly with age in fresh canine semen (Rijsselaere et al. 2007; Tesi et al. 2018; Fuente-Lara et al. 2019). However, one study reported a negative correlation between dog age, sperm concentration (p < 0.01, r = -0.16), and total sperm output (p < 0.0001, r = -0.18) in fresh canine semen (Goericke-Pesch and Failing 2013). This discrepancy could be due to age, breed and nutrition of the dogs.

### Sperm motility, morphology, and viability

In fresh canine semen, the majority of studies showed that sperm motility and viability are not affected by age (Rijsselaere et al. 2007; Tesi et al. 2018; Fuente-Lara et al. 2019). However, one study investigating age-related changes in epididymal sperm showed that sperm motility and viability in senior dogs is significantly lower than the young and adults (Bhanmeechao et al. 2018). Based on a study by Goericke-Pesch and Failing (2013), a negative correlation was observed between dog age and several measures of sperm quality in ejaculated semen. Specifically, they found a significant negative correlation between dog age and total motility (p < 0.01, r = -0.16), percentage of progressively motile sperm (p < 0.01, r = -0.16), and live spermatozoa (p = 0.012, r = -0.13).

Sperm motility and viability were found to be significantly different with age in frozen-thawed and chilled semen (Hesser et al. 2017; Fuente-Lara et al. 2019).

This finding suggests that semen from older dogs is more susceptible to cryodamage than semen from young dogs, thus, it is important to consider the age of the dog when cryopreserving semen (Fuente-Lara et al. 2019).

All studies that have examined aging in dogs have reported a significant decrease in the number of morphologically normal spermatozoa as dogs get older ( Rijsselaere et al. 2007; Hesser et al. 2017; Tesi et al. 2018). Similar to other species, the reduced number of normal spermatozoa with advancing age might be the result of incomplete or disturbed spermatogenesis in the seminiferous tubules, testicular degeneration, or an increased number of immature spermatids (James and Heywood 1979; Lowseth et al. 1990; Rijsselaere et al. 2007).

### Sperm chromatin integrity

To our knowledge, two studies have assessed the relationship between sperm chromatin structure and aging in dogs (Hesser et al. 2017; Bhanmeechao et al. 2018). One study reported no significant difference in epididymal sperm DNA fragmentation index (DFI) between age groups in different breeds of dogs (Bhanmeechao et al. 2018). However, using ejaculated spermatozoa, significant difference in sperm DFI has been reported between middle-aged (4–6 years) and old (≥ 7) Labrador retriever dogs (Hesser et al. 2017). 

Studies that evaluated reactive oxygen species (ROS) production and aging in dogs found no statistical difference between age groups (Hesser et al. 2017; Fuente-Lara et al. 2019). Additionally, lipid peroxidation of sperm cell was not significantly different between age groups in dogs (Hesser et al. 2017). To gain a better understanding of the effects of aging on oxidative stress status in dogs, further studies could be conducted that specifically o investigate spermatozoa or seminal plasma oxidative stress status in senior dogs.

## Effect of the age on semen quality of the stallion

In horses, unlike in other domestic livestock species, stallions are seldom selected for breeding based on their reproductive performance. Stallions, in particular, are usually chosen based on their physical characteristics, pedigree, or athletic performance, rather than their reproductive abilities (Brito 2007). In recent times, only afew studies have examined age as a possible source of variation in the semen quality of stallions (Waheed et al. 2015). Furthermore, the majority of studies have only investigated the influence of age on basic semen characteristics, while the molecular and biochemical mechanisms of sperm cell aging in stallions have yet to be fully explored.

Typically, sperm quality in stallions of various breeds starts to increase from 4 years of age, peaks at around 12 years, and then begins to decline at approximately 13 years (Darr et al. 2017).

### Semen volume, sperm concentration and total number of sperm per ejaculate

Compared to other domestic animal species, stallions have a longer lifespan and breeding lifespan. As a result, they reach sexual maturity at a later age, typically around five years old (Darr et al. 2017). Semen volume (total ejaculate, gel, and gel-free fractions) tends to increase with age in stallions. Colts (2 to 3 years) and senior stallions (older than 14 years) were found to produce significantly smaller amounts of semen compared to adult stallions (Squires et al. 1979; Dowsett and Knott 1996).

Sperm concentration and total number of spermatozoa in horses of various breeds was reported to be significantly lower in colts less than 3 years old compared to older stallions aged 5 to 13 (Dowsett and Knott 1996). Like in other domestic animals species, the effects of age on sperm quality in stallion are related to immature spermatogenesis in colts, decline in epididymal function and testicular degeneration due to aging (Dowsett and Knott 1996).

### Sperm motility, morphology, and viability

Total sperm motility and percentage of normal sperm was shown to be affected by age in the stallion. Adult stallions aged 5 to 13 years were foundto have significantly higher sperm motility and normal spermatozoa compared to young stallions (≤ 3 years) and senile stallions (≥ 14 years) (Dowsett and Knott 1996).

One study reported that mitochondrial reactive oxygen species (ROS) production in stallion sperm increased continuously (25% to 40%) from 4 to 19 years of age (Darr et al. 2017). It was suggested that the result might be due to reduced mitochondrial turnover within the spermatogonia of aging testicle (Darr et al. 2017). They reported that ROS production is negatively correlated with sperm motility in stallions.

## Conclusion and prospects

**Fig. 2:**
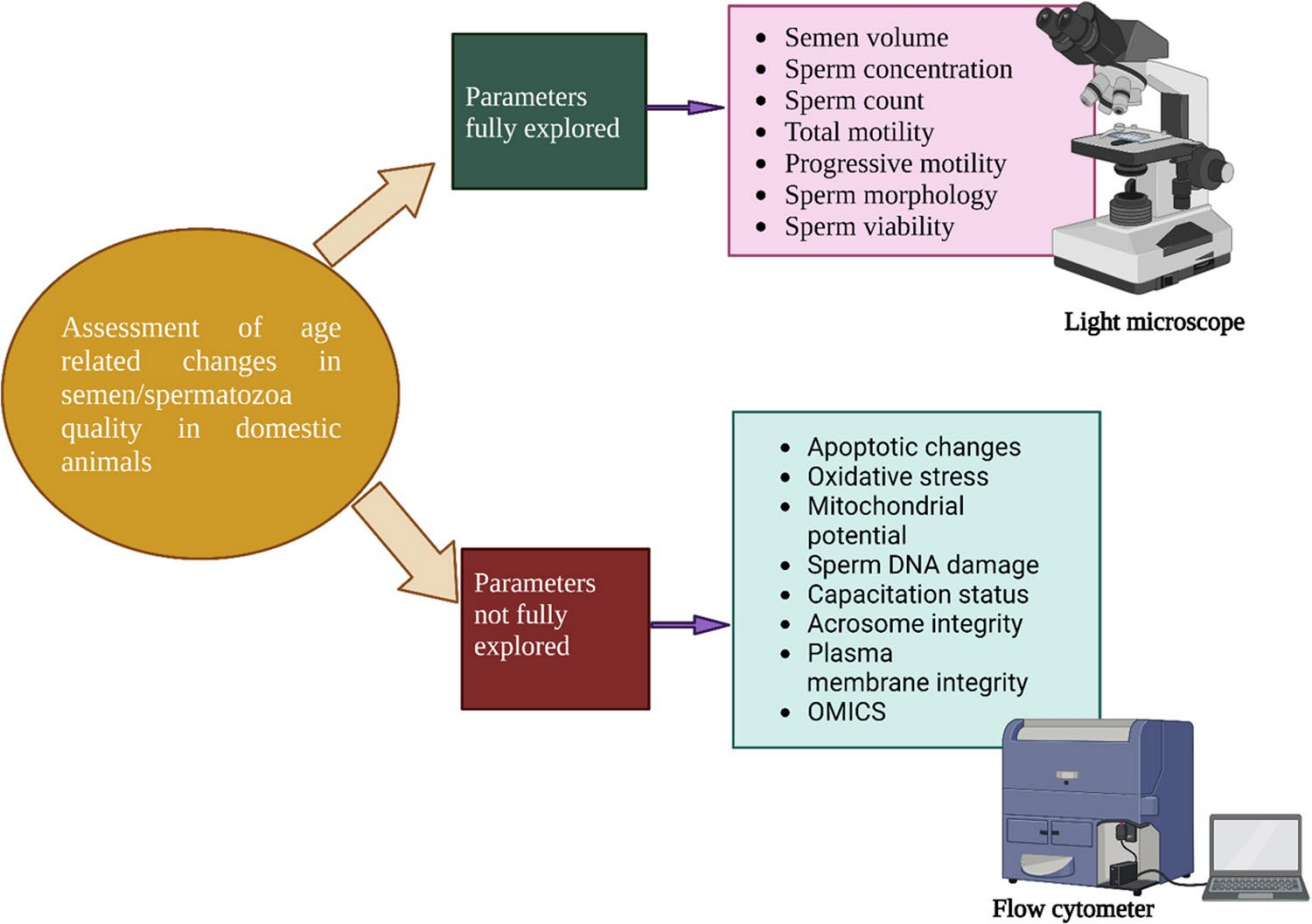
Possible gaps in domestic animal age-related changes on sperm cell function

Although the inverse relationship between sperm quality and male age in relation to basic semen characteristics has been established, few studies have investigated the relationship between paternal age and advanced sperm functional parameters in domestic animals. These advanced parameters include apoptotic changes, oxidative stress, sperm DNA damage, mitochondrial potential, acrosome integrity, and sperm plasma membrane integrity, which are evaluated using flow cytometry and fluorescent microscopy (Fig. 2). Similarly, only a few studies have examined the relationship between aging and pregnancy outcomes, such as abortion and live birth rates. However, current data generally suggests that sperm quality increases from puberty to adult age, where it gradually begins to decline as the animal gets older and senescent. This decline can affect the fertility status and outcomes of assisted reproductive technologies (ARTs).. The age at which an animal becomes mature, adult, or old differs between different species. Consequently, each species was discussed separately.

Although we have attempted to report findings on the effect of male age on sperm quality in domestic animals, it is important to note that interpreting these data can be challenging and susceptible to complications from many confounding factors. With reference to the confounding factors identified in many studies discussed above, male age effects should be evaluated while also accounting for season of the year and breed of the animal.

Other factors, such as semen type (fresh or frozen), temperature, frequency of semen collection, method of semen collection, operator, normal or abnormal semen parameters and the health status of the animal should also be considered.

Sperm cryopreservation is a potential solution to extend the reproductive use of genetically valuable males. However, the process itself, can negatively impact sperm quality, such as reducing sperm motility, viability, and DNA structure (Bailey et al. 2000; Grötter et al. 2019; Khan et al. 2021). To improve the outcome in older animals, antioxidants likeα-tocopherol (vitamin E) can be used during *In vitro* sperm preparation. Research has shown that antioxidants can enhance sperm quality by improving motility, viability, and DNA quality, which are crucial factors for successful fertilization (Kowalczyk 2021).

To our knowledge, no study has investigated the differences in spermatozoa and seminal plasma OMICS (genomics, proteomics, transcriptomics, metabolomics and lipidomics) between young and old animals. Such studies could provide insights into the mechanism of sperm cell aging. Additionally, no research has linked male age to the risk of developmental abnormalities in the progeny or assessed the impact of feed, epigenetic and endocrine disruptors on sperm quality and fertility in animals of different ages.

Further studies may be needed to assess the effect of advancing age on pregnancy and conception rates through *In vivo* fertility studies. Additionally, the outcome of assisted reproductive technologies such as in vitro fertilization, intrauterine insemination, and embryo transfer needs to be studied in animals of different ages. Conducting such studies in dogs for instance, may contribute to advancing knowledge in human-assisted reproductive techniques used in patients of advanced paternal and maternal age.

Studying groups of young and senior animals, particularly those kept for an extended period like stallions and dogs, can shed light on the mechanism of reproductive aging. This knowledge may contribute to understanding the pathways leading to age-related decline in male human fertility. The relationship between advanced paternal age and the risk of chromosomal disorders such as aneuploidy, diploidy, disomy, and trisomy in spermatozoa and embryos has not been fully elucidated in humans (Halvaei et al. 2020). It may be interesting to see how animal models may be used to answer some questions in this area.

It has been reported that the offspring of fathers of advanced paternal age may be at risk of developing cancers (Halvaei et al. 2020). Similar studies could be conducted in dogs, as cancer is a significant disease in this species. Such findings could provide insight into the link between aging and childhood cancers. Moreover, the stallion or dog could be used as models to investigate the effect of increasing male age on numerous disorders such as achondroplasia, autism, schizophrenia, bipolar disorders, and other diseases observed in children of aged parents.

The studies could be feasible in dogs because it is relatively easy to collect semen samples, create uniform age groups of males, exclude unhealthy individuals to avoid misleading results, and compare age groups using biological tests such as artificial insemination trials.

## Data Availability

Not applicable. The articles supporting the conclusions in this manuscript have been duly cited.
